# Chemokine receptor CXCR7 regulates the invasion, angiogenesis and tumor growth of human hepatocellular carcinoma cells

**DOI:** 10.1186/1756-9966-29-31

**Published:** 2010-04-11

**Authors:** Ke Zheng, Hong-Yuan Li, Xin-Liang Su, Xiao-Yi Wang, Tian Tian, Fan Li, Guo-Sheng Ren

**Affiliations:** 1 Department of Endocrine Surgery and breast cancer centre, the First Affiliated Hospital, Chongqing Medical University, Chongqing, PR China

## Abstract

**Background:**

In spite of recent advances in diagnostic and therapeutic measures, the prognosis of hepatocellular carcinoma (HCC) patients remains poor. Therefore, it is crucial to understand what factors are involved in promoting development of HCC. Evidence is accumulating that members of the chemokine receptor family are viewed as promising therapeutic targets in the fight against cancer. More recent studies have revealed that chemokine receptor CXCR7 plays an important role in cancer development. However, little is known about the effect of CXCR7 on the process of HCC cell invasion and angiogenesis. The aim of this study is to investigate the expression of CXCR7 in hepatocellular carcinoma tissues and cell lines and to evaluate the role of CXCR7 in tumor growth, angiogenesis and invasion of HCC cells.

**Methods:**

We constructed CXCR7 expressing shRNA, and CXCR7shRNA was subsequently stably transfected into human HCC cells. We evaluated the effect of CXCR7 inhibition on cell invasion, adhesion, VEGF secretion, tube formation and tumor growth. Immunohistochemistry was done to assess the expression of CXCR7 in human hepatocellular carcinoma tissues and CD31 in tumor of mice. We also evaluated the effect of VEGF stimulation on expression of CXCR7.

**Results:**

CXCR7 was overexpressed in hepatocellular carcinoma tissues. We showed that high invasive potential HCC cell lines express high levels of CXCR7. *In vitro*, CXCL12 was found to induce invasion, adhesion, tube formation, and VEGF secretion in SMMC-7721 cells. These biological effects were inhibited by silencing of CXCR7 in SMMC-7721 cells. In addition, we also found that VEGF stimulation can up-regulate CXCR7 expression in SMMC-7721 cells and HUVECs. More importantly, enhanced expression of CXCR7 by VEGF was founctional. *In vivo*, tumor growth and angiogenesis were suppressed by knockdown of CXCR7 in SMMC-7721 cells. However, silencing of CXCR7 did not affect metastasis of tumor *in vivo*.

**Conclusions:**

Increased CXCR7 expression was found in hepatocellular carcinoma tissues. Knockdown of CXCR7 expression by transfected with CXCR7shRNA significantly inhibits SMMC-7721 cells invasion, adhesion and angiogenesis. Finally, down-regulation of CXCR7 expression lead to a reduction of tumor growth in a xenograft model of HCC. This study provides new insights into the significance of CXCR7 in invasion and angiogenesis of HCC.

## Background

HCC is one of the common types of cancers worldwide and the incidence of HCC is increasing. Understanding the molecular mechanisms that control HCC provides the foundation for therapeutic intervention. Invasion, angiogenesis and metastasis is a typical process of HCC progression. The process of HCC invasion and metastasis is a multistep event that involves cell migration, local invasion, angiogenesis and growth at a secondary site [1,2]. Angiogenesis plays an important role in tumor progression and the development of metastases, and may be proved to be a useful prognostic biomarker for HCC. Controlling the invasion and angiogenesis of cancer remains a crucial goal for the successful treatment of HCC. The lack of effective therapies for HCC is related to poor understanding of the molecular mechanisms underlying cancer invasion and metastasis. Thus, elucidation of molecular mechanisms related to progression and new biomarkers for the malignant potential of HCC are urgently needed. There is abundant evidence to show that chemokine CXCL12 and its receptors (CXCR4, CXCR7) are involved in progression of tumors [3,4].

Stromal cell-derived factor-1 (SDF-1, also called CXCL12) is a member of the CXC subfamily of chemokines and express in a variety of tissues including lung, liver, bone marrow and lymph nodes [5-7]. CXCL12 elicits biologic function through binding to its receptor, CXCR4, which is present on the cell surface and is a seven-transmembrane span G-protein-coupled receptor [8]. CXCL12 plays a role in a number of important physiological processes including leukocyte trafficking and vasculogenesis [6,9]. More importantly, CXCL12 plays a crucial role in the process of invasion and metastasis of tumor cells [3]. CXCL12 stimulates proliferation, dissociation, migration, and invasion in a wide variety of tumor cells, including breast cancer cells, pancreatic cancer cells and HCC cells [3,10,11].

CXCR4 belongs to the large superfamily of G protein-coupled receptors and plays an important role in a variety of normal cellular processes, such as vascularization, nervous systems development and haematopoiesis [12,13]. Numerous studies have demonstrated that CXCR4 frequently overexpressed in a variety of human tumors, such as breast cancer, prostate cancer and hepatocellular carcinoma [3,14,15]. It has been shown that the overexpression of CXCR4 significantly correlate with metastasis and poor prognosis in different tumor types [16,17]. In addition, inhibition of CXCR4 function by the administration of AMD3100, CXCR4-specific peptide antagonist, can dramatically impair tumor formation and metastasis [18]. Until recently, CXCR4 was considered to be the only receptor for CXCL12. However, a recent study has shown that chemokine receptor CXCR7 can also bind to CXCL12, and it is identified as a second receptor for CXCL12 [19].

Recently, a newly discovered chemokine receptor called CXCR7 has been identified [19]. CXCR7 mediates a broad range of cellular activities, including proliferation, survival, and adhesion by binding with CXCL12 [19]. However, the function of CXCR7 is still unclear and controversial. Some studies suggested that CXCR7 is a non-signaling decoy receptor and can not activate intracellular signaling cascades. Grymula *et al*. [20] found that CXCR7 expressed on rhabdomyosarcoma cells was a signaling receptor and could activate (MAPK)p42/44 and AKT phosphorylation through binding with its ligand. In addition, CXCR7 participated in regulation of rhabdomyosarcoma cell motility, directional chemotaxis, expression of MMPs, and cell adhesion and enhanced *in vivo *metastatic potential of rhabdomyosarcoma cells. Furthermore, CXCR7 as a inclassical chemokine receptor plays an important role in the CXCL12/CXCR4-mediated transendothelial migration (TEM) of human cancer cells [21]. It has been demonstrated that CXCR7 is expressed in variety of tumor cell lines and normal cells including activated endothelial cells, fetal liver cells, T cells, B cells and renal multipotent progenitors [19,22]. Importantly, overexpression of CXCR7 has been observed in various tumors, including breast cancer, lung cancer, prostate cancer and pancreatic cancer [4,23-25]. Miao *et al*. [4] have shown that CXCR7 promotes tumor growth in a mouse model of lung and breast cancers, and that expression of CXCR7 influences experimental lung metastasis. In addition, expression of CXCR7 enhances the cell adhesion, invasion, blood vessel sprout formation *in vitro *and promotes tumor growth *in vivo *[24]. Another study has revealed that CXCR7 mediated proliferation and chemotaxis of tumor cells towards CXCL12 *in vitro*, but no effect of CXCR7 on tumor growth and metastasis was observed *in vivo *[26]. These results provide a reasonable basis to propose that the CXCL12/CXCR7 interaction could play an important role in cancer progression. Although the role of CXCL12 in the promotion of invasive growth is well documented and the intracellular signals triggered by CXCR4 activation have been extensively investigated, the role of CXCL12/CXCR7 axis in regulating tumor growth of HCC is not yet known. In addition, the published evidence is not consistent on whether CXCR7 expression contributes to tumor growth, invasion and metastasis. Thus, it is necessary to further explore the role of CXCR7 in cancer development.

There is increasing evidence that CXCR7 may participate in tumor development. In previous study, CXCR7 was demonstrated to express on a large percentage of tumor -associated blood vessels of human liver HCC [4]. However, the biological significance of CXCL12/CXCR7 interaction in development of HCC is unclear. The present study was undertaken to test the hypothesis that CXCL12/CXCR7 was involved in malignant properties of HCC. We have studied the expression of CXCR7 in hepatocellular carcinoma tissues and cell lines. We have also evaluated the effect of specific inhibition of CXCR7 on CXCL12-induced cell invasion, adhesion and angiogenesis. In addition, we have investigated whether VEGF stimulation affects CXCR7 expression. Finally, we have further analyzed whether inhibition of CXCR7 expression would affect tumor growth and metastasis *in vivo*.

## Methods

### Patients and tumor specimens

Patients underwent surgical resection at the The First Affiliated Hospital, Chongqing Medical University between February 2008 and October 2009. All cases of hepatocellular carcinoma tissues were diagnosed clinically and pathologically. None of the patients had received any preoperative treatments (radiotherapy or chemotherapy). Hepatocellular carcinoma tissues were embedded with paraffin and stored in Department of Pathology, Chongqing Medical University, China. Paraffin-embedded hepatocellular carcinoma specimens were obtained from 35 HCC patients [22 male, 13 female; average age of 52 years (range, 38-68 years)].

### Construction of Small Hairpin RNA plasmid

Knockdown of CXCR7 was achieved by expression of short hairpin RNA (shRNA) from the pGPU6/Neo vector containing the human U6 promoter (GenePharma, Shanghai, China). All DNA oligonucleotides were synthesized by Shanghai Sangon Biological Engineering Technology & Services Co., Ltd. (Shanghai, China). The sequence of the oligonucleotide targeted to CXCR7 is 5'-GCATCTCTTCGACTACTCAGA -3', corresponding to positions 223 to 243 within the CXCR7 mRNA sequence (accession no. NM_020311). The following complementary oligonucleotide encoding short hairpin RNA was designed to knock down CXCR7: (sense) 5'-CAC CGCATCTCTTCGACTACTCAGATTCAAG AGATCTGAGT AGTCGAAGAGATGCTTTTTTG-3' and (antisense) 5'-GATCCAAAAAAGCATCTC TTCGACTACTCAGATCTCTTGAATCTGAGTAGTCGAAGAGATGC-3'. The pGPU6/Neo plasmid was linearized with *Bam*H I and *Bbs *I to permit the insertion of the annealed oligonucleotides. DNA oligonucleotides were annealed by incubating the mixed oligonucleotides in the PCR thermocycler using the following profile: 95°C for 5 min, 80°C for 5 min, 75°C for 5 min and gradually cooled to room temperature. Annealed oligonucleotides were ligated to the *Bbs*I and *Bam*H I sites of the pGPU6/Neo plasmid. The scrambled shRNA was used as a negative control(referred to as "NC" in the text), of which the sequence was 5'-GACGAGCTTCTACACAATCAT-3'. The recombinant constructs were verified by DNA sequencing and by analyzing the fragments generated from digestion with *Bam*H I. The efficiency of knockdown was determined by Western blot and RT-PCR.

### Cell lines and cell culture conditions

Human HCC cell lines HepG2, Hep3B, SMMC-7721 and human umbilical vein endothelial cells (HUVECs) were purchased from Cell Bank of Shanghai Institute of Biochemistry & Cell Biology, Chinese Academy of Sciences (Shanghai, China). Human HCC cell lines MHCC97L, MHCC97H and HCCLM6 were obtained from Liver Cancer Institute and Zhong Shan Hospital of Fudan University, Shanghai, China. MHCC97L, MHCC97H and HCCLM6 were maintained in DMEM (Gibco, USA) supplemented with 10% heat-inactivated FBS (HyClone, USA). HepG2, Hep3B and SMMC-7721 were cultured in an RPMI-1640 (Gibco, USA) medium supplemented with 10% heat-inactivated FBS. HUVECs was maintained in F12 medium containing 10% FBS (HyClone, USA). All the media were supplemented with 100 U/ml penicillin and 100 μg/mL streptomycin (Invitrogen, USA) and maintained in 5% CO_2 _at 37°C.

### Generation of stable transfectants

SMMC-7721 cells were seeded in six-well plates to 80-90% confluence. The cells were transfected with mixtures of shRNA plasmids and Lipofectamine™ 2000 reagent (Invitrogen, USA) according to the manufacturer's instructions. Forty-eight hours after transfection, transfected cells were grown in growth medium containing 0.4 mg/ml G418 (Gibco, USA) for selection. Stable transfectant clones with low expression of CXCR7 were evaluated by RT-PCR and Western blot analysis. Stable transfectants were expanded for subsequent experiments. SMMC-7721 cells transfected by CXCR7shRNA were referred to as CXCR7shRNA cells, while SMMC-7721 cells transfected by scrambled shRNA as NC cells.

### RNA extraction and reverse transcription PCR

Total RNA in HCC cells was extracted using Trizol (Invitrogen, USA). RT-PCR was performed using reverse transcriptase cDNA synthesis kit (Takara, Japan) according to the manufacturer's protocol. One microgram of total RNA was reversely transcribed into cDNA followed by PCR amplification using specific primers: CXCR7, forward (5'-TGGGTGGTCAGTCTCGT-3') and reverse (5'-CCGGCAGTAGGTCTCAT-3'); CXCR4, forward (5'-CCTGAAGTACCCCATCGAGCAC-3') and reverse (5'-ATACCCCTCGTAGATGGGCACA-3'), GAPDH, forward (5'-GAAGGTGAAGGTCGGAGTC-3') and reverse(5'-GAAGATGGTGATGGGATTTC-3'). CXCR7 was amplified by 30 cycles at 94°C for 40 s, 57°C for 30 s, and 72°C for 1 min in order. CXCR4 was amplified by 30 cycles at 94°C for 35 s, 60°C for 30 s, and 72°C for 1 min in order. Both were followed by a 7 min extension at 72°C. PCR products were electrophoresed on 1.5% agarose gel containing ethidium bromide and visualized by UV-induced fluorescence.

### Western blot analysis

For the preparation of lysates, the cells were washed with ice-cold PBS solution and lysed in lysis buffer (50 mM Tris-HCl, pH 7.4, 150 mM NaCl, 1% Nonidet P-40, and 0.1% SDS supplemented with protease inhibitors). Cells were scraped into microcentrifuge tubes and centrifuged at 10,000 × *g *for 15 min at 4°C. The supernatant was collected, and protein concentrations were determined with the Bio-Rad protein assay reagent according to the Bradford method. Samples were subjected to 10% PAGE analysis after they were boiled for 5 min and electrophoretically transferred to polyvinylidene difluoride (PVDF) membranes (Millipore, USA). Blocking was performed in 5% nonfat dried milk in Tris-buffered saline containing 0.1% Tween 20 at room temperature for 1 h. Membranes were then incubated with primary antibody under constant agitation at antibody dilutions suggested by the antibody supplier overnight at 4°C. After several washings, membranes were incubated with horseradish peroxidase-conjugated secondary antibody (anti-rabbit) for 1 h at room temperature under constant agitation. Proteins were visualized by using an enhanced chemiluminescence system (ECL; Amersham Biosciences, USA).

### Cell invasion assay

SMMC-7721 cells invasion in response to CXCL12 was assayed in the Biocoat Matrigel invasion chamber (Becton Dickinson, USA) with 8-μm porosity polycaronate filter membrane that was coated with Matrigel. Control, NC and CXCR7 shRNA transfected cells were suspended at 3 × 10^5 ^cells/ml in serum-free media respectively, and then 0.2 ml cell suspension was added to the upper chamber. Next, 0.5 ml serum-free media with various concentrations of CXCL12 (0, 10 or 100 ng/ml) was added to the lower chamber. The chambers were then incubated for 24 h at 37°C with 5% CO_2_. After incubation, noinvasive cells were gently removed from the top of the Matrigel with a cotton-tipped swab. Invasive cells at the bottom of the Matrigel were fixed in 4% paraformaldehyde and stained with hematoxylin. The number of invasive cells was determined by counting the hematoxylin-stained cells. For quantification, cells were counted under a microscope in five fields (up, down, median, left, right. ×200).

### Cell adhesion assay

Cell adhesion assay was carried out by using the CytoSelect™ ECM Cell Adhesion Assay kit (Cell BioLabs, USA) following the instruction manual. Briefly, the 48-well plate precoated with laminin (LN) or fibronectin (FN) were washed with PBS twice and blocked for 1 h at 37°C with RPMI 1640 containing 0.1% BSA before plating cells. Plates were again washed with PBS and air-dried. SMMC-7721 cells were preincubated with CXCL12 (100 ng/ml) for 24 h at 37°C. A cell suspension containing 2 × 10^5 ^cells/ml was prepared in serum free media. The cell suspension (150 μl) was added to the inside of each well (BSA-coated wells were provided as a negative control). Cells were allowed to attach for 1 h at 37°C. Subsequently, unattached cells were removed by gentle washing 3 times with PBS. Then the attached cells were stained with 1% crystal violet. Each well was gently washed 3 times with PBS. The total crystal violet bound to the cells was eluted with 10% acetic acid and measured by the absorbance at 560 nm. All the experiments were repeated 3 times in duplicate wells.

### ELISA for VEGF

SMMC-7721 cells were plated in 24-well tissue culture plates at a density of 1 × 10^5 ^cells per well and followed with serum starvation for 24 h with RPMI-1640. Then, cells were treated with recombinant human CXCL12 (100 ng/ml)(Peprotech, UK), and the supernatants were collected 24 h after treatment. VEGF concentration was determined using Quantikine ELISA kits according to the manufacturer's instructions (R&D Systems, Minneapolis, MN).

### In vitro tube formation coculture assay

To perform the tube formation assay, Transwell chambers were precoated with growth factor-reduced Matrigel (200 μL of 10 mg/mL). Control, NC and CXCR7 shRNA transfected cells were seeded at a density of 2 × 10^4 ^cells/well in 24-well plates and cultured for 24 h respectively. HUVECs (2 × 10^4 ^cells/well) were then seeded in Transwell chambers precoated with the Matrigel. Subsequently, Transwell chambers containing HUVECs were inserted into the 24-well plates and cocultured for 24 h. After 24 h of cocultured at 37°C and 5% CO_2_, the number of capillary-like tubes from three randomly chosen fields was counted and photographed under an Nikon inverted microscope (Japan).

### Immunohistochemistry and quantitation of microvessel density

Immunohistochemistry was used to analyze the expression of CXCR7 and CD31. Paraffin-embedded human hepatocellular carcinoma tissues were sectioned at 5 μm thickness. Tumors established in nude mice were isolated and fixed in 4% paraformaldehyde, embedded in paraffin, and cut in 6 μm sections. Tumor sections were deparaffinized, rehydrated, and quenched with 3% hydrogen peroxide for 10 min at room temperature. The sections were incubated in protein blocking solution (5% normal horse serum, 1% goat serum in PBS) for 10 min before the addition of the primary antibody. The sections were incubated for 2 h at 37°C with rat antimouse CD31 (BD Biosciences, USA) or rabbit antihuman CXCR7 (Abcam, UK) at 1:100 dilutions. After incubation, the sections were washed in PBS for 10 min, and anti-mouse or anti-rabbit secondary biotinylated antibody was applied. After washings, the avidin-biotin complex was then applied to the sections, followed by extensive washing steps. Diaminobenzidine chromogen was then added to the sections and incubated in the dark for 5 min.

MVD in tumor tissues was determined by immunohistochemical staining with an endothelial-specific antibody CD31. For Quantitative analyses of MVD, three random high-power fields (×200) were photographed for each tumor section. MVD was calculated as mean number of tumor vessels per high-power field.

### In vivo tumorigenicity

Male nude mice (BALB/c) of six-week-old were purchased from the Laboratory Animal Center of Chongqing Medical University (Chongqing, China) and bred under specified pathogen-free conditions. The mice were randomly divided into three groups composed of five animals each. The control, NC and stable CXCR7shRNA transfected SMMC-7721 cells (1 × 10^6 ^for each) were inoculated subcutaneously into the back of nude mice and tumor size was measured every 4 days. The tumor size was measured by a caliper, and the tumor volume was calculated using the formula (length × width^2^)/2. The mice were sacrificed 32 days after inoculation. The tumors were weighed and fixed in 4% polyformaldehyde. The tumor sections were excised for immunohistochemical analysis.

Tumors dissected from CXCR7shRNA transfected cells were referred to as CXCR7shRNA tumors, while tumors dissected from control and NC cells as control tumors and NC tumors respectively.

### Statistical analysis

Data are reported as means ± SD. The one-way ANOVA was used for data analysis. All statistics were calculated using SPSS 16.0 software (SPSS, Chicago, IL, USA). *P *< 0.05 was considered as statistically significant.

## Results

### Expression of CXCR7 in hepatocellular carcinoma tissues from patients

**Figure 1:**
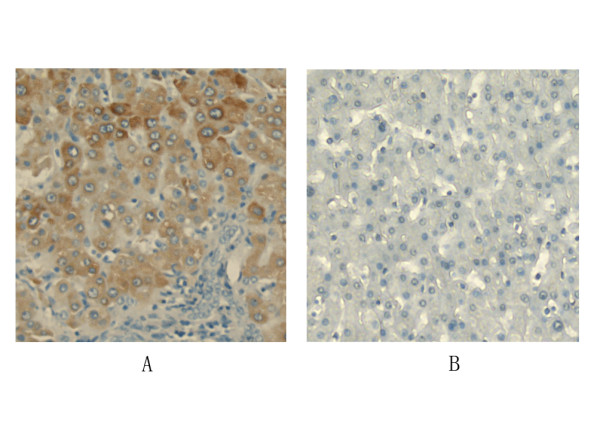
**CXCR7 expression in human hepatocellular carcinoma tissues and normal liver tissues**. Expression of CXCR7 was analyzed in 35 hepatocellular carcinoma and 25 normal liver tissues by immunohistochemistry. Representative pictures of histological sections of both hepatocellular carcinoma (A) and normal liver tissues (B) stained with anti-CXCR7 antibody. Original magnification, 200×.

Little is known about the expression of CXCR7 in HCC. To investigate whether CXCR7 might play a role in HCC development, we first examined its expression in 35 hepatocellular carcinoma tissues and 25 normal liver tissues using immunohistochemistry. The positive ratio of CXCR7 was 91% (32 of 35 cases) in hepatocellular carcinoma tissues. In most cases, the CXCR7 staining localized to both the cytoplasm and the cell membrane but not in the cellular nucleus (Fig. 1A). However, the positive ratio of CXCR7 was only 10% (3 of 25 cases) in normal liver tissues. Most of normal liver tissues displayed very low or undetectable CXCR7 levels (Fig. 1B). Together, these data demonstrated a significant increase of CXCR7 expression level in hepatocellular carcinoma tissues.

### Expression of CXCR7 on HCC cell lines and HUVECs

**Figure 2:**
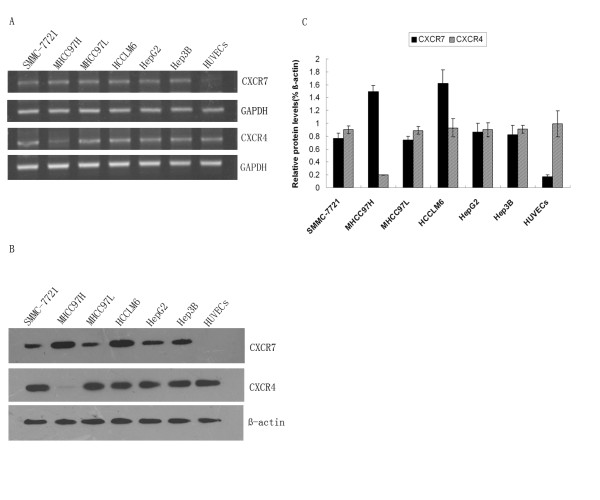
**Expression of CXCR4 and CXCR7 in HCC cell lines and HUVECs**. A. RT-PCR was performed on various cell lines to determine CXCR7 and CXCR4 mRNA expression. GAPDH was used as a control. B. Western blot analysis was performed to detect CXCR7 and CXCR4 protein expression. β-actin was used as a control to ensure equal loading. Data shown is representative of three independent experiments. C. The intensity of protein bands was quantified and was shown as relative expression level after normalized by β-actin (n = 3, means ± SD).

Initial evidence has indicated that CXCR7 is overexpressed in many human cancer cells [4,24,25]. To determine whether CXCR7 is also expressed in HCC cell lines, we first evaluated the expression of CXCR7 by RT-PCR in a panel of HCC cell lines (HepG2, Hep3B, SMMC-7721, MHCC97L, MHCC97H and HCCLM6). As shown in Fig. 2A, CXCR7 mRNA expression was clearly detected in six HCC cell lines, with different amounts of CXCR7 transcripts; in particular, the expression of CXCR7 was the highest in MHCC97H and HCCLM6 cells. In addition, most of the HCC cell lines expressed both of the CXCR7 and CXCR4 (Fig. 2A). Expression of CXCR7 mRNA was also tested in HUVECs. We observed low levels of CXCR7 mRNA expression in HUVECs (Fig. 2A).

To determine CXCR7 protein expression, Western blot analysis was conducted on protein samples derived from HUVECs and a panel of HCC cell lines. The results of Western blot analysis are similar with RT-PCR analysis. As shown in Fig. 2B and 2C, all HCC cell lines expressed CXCR7. All low aggressive cell lines (HepG2, Hep3B, SMMC-7721 and MHCC97L) had lower levels of CXCR7. In HUVECs, CXCR7 was almost undetectable. Of interest, the high aggressive cell lines (MHCC97H and HCCLM6 cells)exhibited higher levels of CXCR7 protein than did the low aggressive cell lines. These results imply the potential involvement of CXCR7 in invasion of cancer cells.

### The vector stably expressing CXCR7shRNA causes effective and specific down-regulation of CXCR7 expression

**Figure 3:**
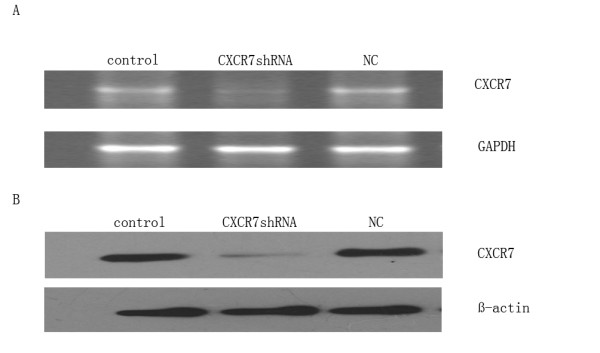
**Downregulation of CXCR7 expression in SMMC-7721 cells by transfection with CXCR7shRNA**. SMMC-7721 cells were stably transfected with CXCR7shRNA. CXCR7 expression was strongly suppressed by specific CXCR7shRNA. A. cellular RNA was harvested after G418 selection and CXCR7 mRNA was measured using RT-PCR. GAPDH was used as a loading control. B. after G418 selection, the protein expression levels of CXCR7 were measured by Western blot using anti-CXCR7 antibody and β-actin as a loading control. The experiment was repeated three times with similar results.

In order to study the potential role of CXCR7 in HCC cell lines, we used pGPU6/Neo-shCXCR7 directed at nucleotides 223 to 243 of CXCR7 to selectively reduce CXCR7 expression in the SMMC-7721cells. CXCR7shRNA and scrambled shRNA were used to transfect SMMC-7721 cells. After G418 selection, the knockdown efficiencies were subsequently tested using RT-PCR and Western blot. As shown in Fig. 3A, CXCR7 mRNA levels were reduced by 85.0% in CXCR7 shRNA transfected cells, compared with the control cells. Similar to RT-PCR results, the expression level of CXCR7 protein were reduced by 80.0% in CXCR7 shRNA transfected cells (Fig. 3B). The scrambled sequence shRNA had no effect on CXCR7 expression (Fig. 3B). These results demonstrated that the expression of CXCR7 was specifically silenced in SMMC-7721 cells.

### CXCR7 silencing inhibits CXCL12 induced enhancement on HCC cells invasion in vitro

**Figure 4:**
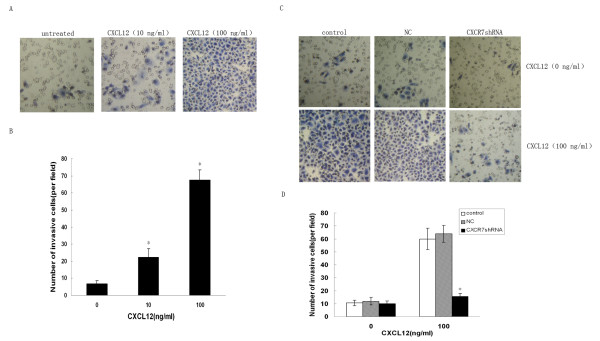
**silencing of CXCR7 inhibits CXCL12 induced enhancement on SMMC-7721 cells invasion *in vitro***. A. SMMC-7721 cells were examined for their invasive ability after stimulation with different concentrations of CXCL12 (0, 10 or 100 ng/ml). Representative pictures are shown. B. mean number of invasive cells from each group. Data are expressed as means ± SD. *p < 0.05 (as compared with untreated cells). C. CXCR7shRNA transfected, NC and control cells were treated with CXCL12 (100 ng/ml). The invasive ability of CXCR7shRNA transfected cells appeared significantly reduced, compared with control cells and NC cells. The pictures highlight the differences in number between the CXCR7shRNA transfected, control and NC cells able to invade through Matrigel. D. mean number of invasive cells from five independent fields/well is indicated. Data are expressed as means ± SD from three independent experiments. *p < 0.05 (as compared with control cells).

The CXCL12/CXCR7 interaction was reported to regulate invasive and metastatic behavior of several tumors [4,24]. It is therefore of interest to investigate the effect of CXCR7 on HCC cells invasion by reducing CXCR7 expression using siRNA. To evaluate a role of CXCR7 in regulating the invasive ability of HCC cells, we selected the SMMC-7721 cell line as a model. Cell invasion experiments were performed with a Matrigel invasion chamber, which is considered an *in vitro *model system for metastasis. As shown in Fig. 4A and 4B, SMMC-7721 cells spontaneously invaded through artificial basement membrane in the absence of CXCL12. In addition, we found that CXCL12 induced a significant and dose-dependent increase of cancer cell invasion through Matrigel. We next evaluated the effect of silencing of CXCR7 on SMMC-7721 cells invasion. The CXCR7shRNA cells displyed decreased invasive ability compared with control cells and NC cells (Fig. 4C and 4D). Taken together, these findings indicate that CXCL12 potently enhances the invasive ability of SMMC-7721 cells and that silencing of CXCR7 inhibits the invasive behavior of the cells induced by CXCL12.

### CXCR7 silencing inhibits CXCL12 induced enhancement on HCC cells adhesion in vitro

**Figure 5:**
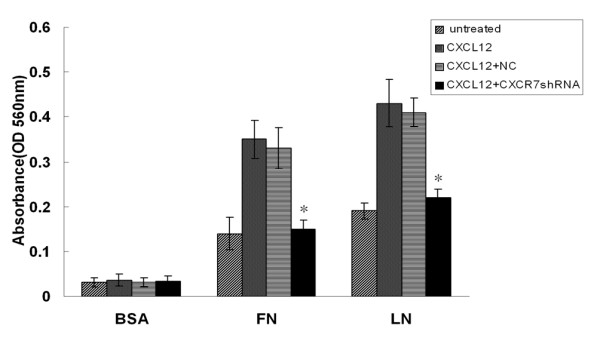
**Effect of CXCR7 silencing on HCC cells adhesion *in vitro***. SMMC-7721 cells were treated as described in Materials and Methods. SMMC-7721 cells displayed an enhanced cell adhesion to LN or FN in the presence of CXCL12. Cells transfected with CXCR7shRNA showed significantly reduced ability of adhesion to LN or FN compared with control and NC cells. Each bar represents mean ± SD from three independent experiments. *p < 0.05 (as compared with untransfected cells).

Tumor cell adhesion to the ExtraCellular Matrix (ECM)is an important step of the invasion process. To analyze the effect of CXCR7 expression on the adhesion of tumor cells to LN or FN, HCC cells were examined by a cell adhesion assay. As shown in Fig. 5, SMMC-7721 cells displayed an enhanced cell adhesion to LN or FN in the presence of CXCL12. Adhesion of SMMC-7721 cells to LN was greater than adhesion to FN or BSA. However, cells transfected by CXCR7shRNA showed significantly reduced ability of adhesion to LN or FN compared with the control and NC cells. Control, NC and CXCR7shRNA transfected cells adhered equally to BSA-coated dishes. Together, these results indicate that treatment with CXCL12 increases adhesive ability of SMMC-7721 cells and CXCR7 silencing results in decreased adhesive ability.

### CXCR7 silencing inhibits tumor cell-induced tube formation in vitro

**Figure 6:**
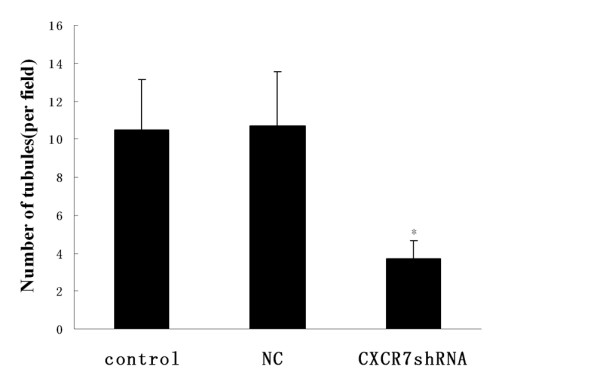
**Effect of CXCR7 silencing on tube formation induced by SMMC-7721 cells**. HUVECs were cocultured with SMMC-7721 cells, as described in Materials and Methods. Inhibition of CXCR7 expression in SMMC-7721 cells impaired tube formation induced by SMMC-7721 cells. Each bar represents mean ± SD from three independent experiments. *p < 0.05 (as compared with control cells).

To address whether CXCL12/CXCR7 interaction could mediate *in vitro *tumor cell-induced tube formation, a coculture system was used in which HUVECs were induced by HCC cells to form capillary-like structures. The tube formation of HUVECs on the Matrigel was quantified by measuring the tube number. As shown in Fig. 6, control and NC cells induced HUVECs to differentiate into capillary-like structures within 24 h. In contrast, SMMC-7721 cells transfected with CXCR7shRNA markedly inhibited tumor cell-induced tube formation. HUVECs showed a significant 32% decrease in the number of tubes after transfecting SMMC-7721 with CXCR7shRNA.

### CXCL12 induces VEGF secretion through CXCR7 in HCC cells

**Figure 7:**
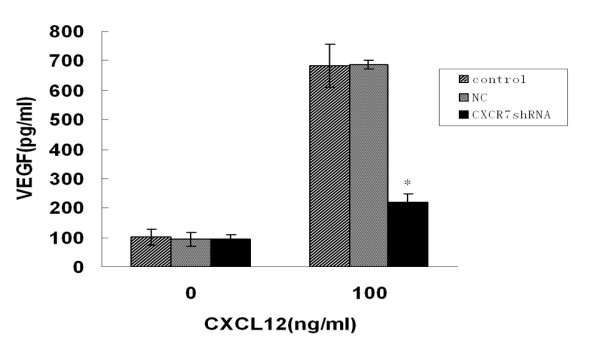
**CXCL12 induces VEGF secretion through CXCR7 in SMMC-7721 cells**. SMMC-7721 cells were plated in the 24-well plates. SMMC-7721 cells were serum starved for 24 h, and the cells were treated with CXCL12 (100 ng/ml). The cultured supernatants were harvested 24 h after treatment, and VEGF was measured by ELISA assay. VEGF secretion of SMMC-7721 cells increased significantly after treatment with CXCL12 for 24 h. Cells transfected with CXCR7shRNA displayed decreased VEGF secretion compared with control and NC cells. Each bar represents mean ± SD from three independent experiments. *p < 0.05 (as compared with control cells).

To evaluate whether CXCL12 contributes to proangiogenic factor secretion in HCC cells, we treated SMMC-7721 cells with CXCL12 (100 ng/ml) and measured secretion of proangiogenic factor VEGF by ELISA analysis. As shown in Fig. 7, VEGF secretion increased significantly when SMMC-7721 cells were treated with CXCL12 for 24 h. To further investigate whether VEGF secretion was mediated by CXCR7, CXCR7 expression was inhibited by RNA interference before treatment with CXCL12. Significant reduction in VEGF secretion was observed in CXCR7shRNA cells compared with control and NC cells. Thus, these findings indicate that CXCL12 can induce VEGF secretion in SMMC-7721 cells and that CXCR7 can serve as a factor involved in regulation of secretion of VEGF.

### CXCR7 is up-regulated by VEGF stimulation and enhances HCC cells invasion

**Figure 8:**
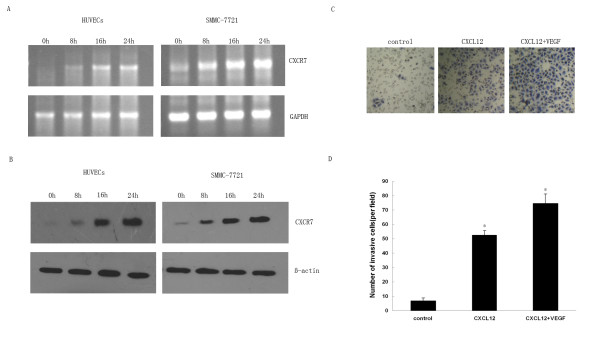
**Effect of VEGF stimulation on CXCR7 expression in HUVECs and SMMC-7721 cells**. HUVECs and SMMC-7721 cells were stimulated for 8, 16 and 24 h in the presence or absence of VEGF (50 ng/ml) respectively. A. total RNA was analyzed by RT-PCR for CXCR7 mRNA expression. GAPDH was used as an internal control. B. HUVECs and SMMC-7721 cells were treated as in A and then subjected to Western blot analysis to examine CXCR7 protein expression. β-actin was used as an internal control. Results are representative of three separate experiments. C and D. SMMC-7721 cells pretreated or not with VEGF (50 ng/ml) were used for Matrigel invasion assay, adding CXCL12 (100 ng/ml) to the bottom chamber. The number of invasive cells in five fields/well is reported. Data are expressed as means ± SD from three independent experiments.*p < 0.05 (as compared with untreated cells).

Burns *et al*. [4] have shown that CXCR7 expression can be up-regulated by TNF-α and IL-1β stimulation. To explore whether expression of CXCR7 could be affected by VEGF simulation, we first used PT-PCR analysis to evaluate the effect of VEGF (50 ng/ml) on CXCR7 expression in HUVECs and SMMC-7721 cells. Interestingly, we found that VEGF substantially increased CXCR7 mRNA in a time-dependent manner (Fig. 8A). In HUVECs, the CXCR7 mRNA increased as early as 8 h after VEGF treatment and showed further up-regulation at 16 h and 24 h. VEGF treatment of SMMC-7721 cells also caused an increase in CXCR7 mRNA in a time-dependent manner starting as early as 8 h.

We also tested CXCR7 protein expression with Western blot analysis. Consistent with the RT-PCR results, CXCR7 protein levels were time-dependently increased after VEGF stimulation (Fig. 8B). In HUVECs, CXCR7 protein levels were changed at 8 h and significantly increased at 16 h and 24 h following VEGF stimulation. When SMMC-7721 cells were treated with VEGF, CXCR7 protein levels increased starting at 8 h and peaked at 24 h. Earlier studies have shown CXCR7 frequently overexpressed on tumor blood vessels [4]. One possible explanation might be that cytokines such as, TNF-α, IL-1β and VEGF produced from tumor microenvironment enhanced the expression of CXCR7.

To further evaluate whether the up-regulation of CXCR7 expression by VEGF stimulation is functional, Matrigel invasion assay was performed to analyze the effect of VEGF on the invasion of the HCC cells towards CXCL12. SMMC-7721 cells pretreated with VEGF for 16 h were allowed to invade through a Matrigel-coated membrane towards CXCL12 for 24 h. Although SMMC-7721 cells demonstrated invasive ability in response to CXCL12 in the abesence of VEGF, the magnitude of these responses was significantly enhanced in those cells pretreated with VEGF for 16 h (Fig. 8C and 8D). Therefore, we have demonstrated that VEGF treatment not only increases expression of CXCR7 on SMMC-7721 cells but also enhances the invasive ability of these cells in response to CXCL12.

### Inhibition of tumor growth and angiogenesis by silencing of CXCR7

**Figure 9:**
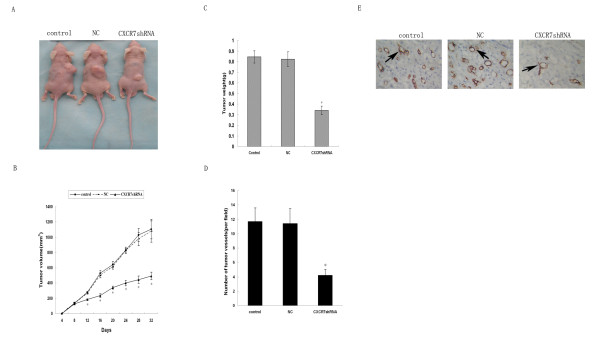
**Effect of CXCR7 silencing on tumor growth**. About 2 × 10^6 ^CXCR7shRNA, control and NC cells were inoculated subcutaneously into the back of five different nude mice in each group. On day 32 after tumor inoculation, the mice were sacrificed. A. representative pictures from each group of mice bearing tumors. B. tumor volume was measured at the indicated days. Data shown are means ± SD (n = 5). *p < 0.05 (as compared with both control and NC tumors). C. weight of the tumor was determined after dissection at the end of the experiment. As shown, both tumor volume and tumor weight were dramatically decreased as the consequence of CXCR7 silencing. Data shown are means ± SD (n = 5). *p < 0.05 (as compared with both control and NC tumors). D. tumor sections were examined for MVD. Tumor vessels in a three randomly selected fields were counted in tumor sections in each group. Data shown are means ± SD. *p < 0.05 (as compared with control and NC tumors). E. inhibition of tumor angiogenesis by silencing CXCR7. Tumor sections were stained with anti-CD31 antibodies. Positive staining is indicated by an arrow.

The results of *in vitro *studies strongly suggested that CXCR7 mediated invasion and angiogenesis. To investigate whether CXCR7 plays a role in tumorigenesis, we inhibited expression of CXCR7 by transfecting SMMC-7721 cells with CXCR7shRNA. After G418 selection, CXCR7shRNA, NC and control cells were inoculated subcutaneously into the back of nude mice and tumor size was measured every 4 days. Interestingly, tumor growth was affected by knockdown of CXCR7 expression in SMMC-7721 cells. As shown in Fig. 9A, B and 9C, SMMC-7721 cells transfected with CXCR7shRNA showed significantly reduced tumor growth compared with control and NC cells. At the end of 32 days, control tumors grew to an average size of 1107.6 ± 128.3 mm^3 ^and 0.845 ± 0.057 g. CXCR7shRNA tumors grew to 493.8 ± 49.6 mm^3 ^and 0.341 ± 0.039 g, showing 55.3% tumor growth inhibition which is statistically different from control tumors. No statistic differences were obtained between NC tumors and control tumors. No weight loss and decreased activity were observed in all the mice (data not shown). Therefore, these results indicate that silencing of CXCR7 substantially inhibited the tumor growth.

The above data demonstrated that silencing of CXCR7 substantially suppressed tumor growth. One possible mechanism for slower growth of CXCR7shRNA tumors was the decreased angiogenesis. Therefore, we investigated whether silencing of CXCR7 could result in suppression of tumor angiogenesis. MVD was calculated by averaging the CD31^+ ^microvessels of tumors in each group. As shown in Fig. 9E, tumor vessels formation was suppressed in CXCR7shRNA tumors. Silencing of CXCR7 resulted in a significant reduction of MVD in CXCR7shRNA tumors compared with those of control and NC tumors (Fig. 9D). These results indicated that silencing of CXCR7 substantially suppressed angiogenesis and subsequently inhibited the tumor growth.

To extend our *in vitro *findings and evaluate the contribution of CXCR7 to metastasis formation *in vivo*, the effect of CXCR7 silencing on organ metastasis was next examined. We did not observe that HCC cells spontaneously metastasized to the lungs and other organs of mice (data not shown). None of the mice developed lung metastasis.

In summary, results from the heterotopic models showed that silencing of CXCR7 inhibited the tumor growth but not the metastasis of HCC cells *in vivo*.

## Discussion

The identification of new biomarkers for the early detection of HCC is critical in the development of tumor-targeted therapy, and would likely have an important positive effect on the prognosis of this disease. CXCL12 plays a well-recognized role in the process of tumor progression. Accumulating evidence indicates that CXCL12 and its receptors are involved in cancer development through the inhibition of apoptosis, and promotion of angiogenesis, cellular proliferation, invasion and metastasis [27,28]. CXCR7 expression has been reported in various human cancers, including carcinomas of the lung, prostate, pancreas and breast, as well as HCC [4,23-25]. In the present study, we observed that human hepatocellular carcinoma tissues exhibited increased expression of CXCR7 as compared to normal liver tissues. We also found that expression of CXCR7 is elevated in all six HCC cell lines compared with HUVECs. In addition, we observed that high metastatic potential cell lines expressed significantly higher levels of CXCR7 than low metastatic potential cell lines. This finding implies that CXCR7 overexpression may be involved in invasion and metastasis nature of HCC.

Considerable efforts have been made in recent years to elucidate the biological function of chemokine receptors in cancer invasion and metastasis. To date, the role of CXCR7 in regulating HCC cells invasion is unclear. In this study, we observed that treatment with CXCL12 enhanced invasion and silencing of CXCR7 significantly inhibited the invasive ability of SMMC-7721 cells. Our study indicated the significance of CXCR7 on HCC cells invasion. These results are consistent with recent studies showing that CXCR7 mediates chemotaxis of cancer cells towards CXCL12 [24,26]. Some studies have shown that CXCR7 can not trigger chemotaxis and activate calcium mobilization and intracellular signaling cascades, such as PI3K and ERK pathways [19,29]. However, a recent study has demonstrated that CXCR7 is not a decoy but a functional seven-transmembrane span chemokine receptor and can induce phosphorylation of MAPK p42/44 and AKT in human rhabdomyosarcoma cell lines [20]. In this study, we did not elucidate the molecular mechanisms by which CXCR7 regulated the invasion of HCC cells. Another recent study suggests that signaling pathways mediated by CXCR7 are independent of those triggered through CXCR4 [30]. Therefore, it is reasonable to speculate that CXCR7 may exert effects on other signaling. Also, the different biological effects elicited by CXCR7 may depend on cell type. Thus, further studies elucidating roles of CXCR7 in invasion and signaling cascades activated by CXCL12/CXCR7 axis are required.

Tumor cells interact with ECM components and basement membranes, an essential initial event during the process of invasion. It also has been reported that expression of CXCR7 can regulate adhesion of tumor cells to endothelial cells [19,24]. Our results demonstrated that CXCL12 could induce adhesion of SMMC-7721 cells to FN and LN. The enhanced cell-matrix adhesion may contribute to metastasis of tumor cells. In addition, we also found that RNAi-mediated down-regulation of CXCR7 significantly inhibited CXCL12 induced adhesion of SMMC-7721 cells to LN or FN. Therefore, these findings clearly indicate that CXCR7 participate in CXCL12 induced cell-matrix adhesion. Tumor metastasis is a multistep process that involves the coordinated events of invasion, adhesion, proteolysis and migration. The decreased adhesive ability of HCC cells could lead to inhibition of the invasion of SMMC-7721.

Cancer cells depend on angiogenesis to survive and proliferate [31]. We observed that HCC cells could induce *in vitro *tube formation, which could promote tumor growth. Although CXCL12 induced VEGF secretion has been reported in various cells, such as lymphohematopoietic cells and prostate cancer cells [32,33], CXCL12 induced VEGF production in HCC cells has not been previously studied. In the current study, we found that CXCL12/CXCR7 interaction promoted secretion of VEGF, a potent survival factor for endothelial cells, and one of the most prominent angiogenic factors produced by various tumor cells. Furthermore, our data demonstrate that knockdown of CXCR7 inhibits secretion of VEGF and tube formation, suggesting that CXCR7 may be involved in the regulation of angiogenesis in HCC.

Initial evidence has indicated that expression levels of CXCR7 are frequently high in tumor-associated endothelial cells and activated endothelial cells, but not in normal endothelial cells [4,19]. Our results also confirm that CXCR7 expresses in HUVECs with low levels. To date, very little is known in regard to the regulation of CXCR7 expression in cancer cells and normal cells. In this study, we demonstrated that VEGF stimulation enhanced CXCR7 mRNA and protein levels not only in HCC cell lines but also in HUVECs. A large quantity of VEGF is produced from tumor microenvironment, which could result in enhanced expression of CXCR7 in tumor-associated blood vessels. Further studies elucidating molecular mechanisms by which VEGF regulates expression of CXCR7 are under way. Interestingly, we also observed that invasive ability of SMMC-7721 cells pretreated with VEGF was significantly enhanced. These results clearly indicated that VEGF-induced expression of CXCR7 in HCC cells was functional. Because VEGF is a secreted mitogen and plays a key role in regulating tumor angiogenesis [34], we can assume that under pathological conditions such as cancer, CXCR7 may be up-regulated by VEGF and that CXCR7, in turn, might exert an angiogenic effect increasing VEGF production through the CXCL12/CXCR7 axis.

Previous reports have demonstrated that CXCR7 plays an important role in tumor growth [4,19,24]. However, the data from Meijie *et al*. [29] have shown no effect of CXCR7 on tumor growth and metastasis was observed. One possible explanation might be that the different effects of CXCR7 on tumor growth and metastasis may be dependent on cell type. To further confirm our *in vitro *findings, we have explored the role of CXCR7 in tumor growth in SMMC-7721 xenograft mouse tumor model. In the present study, RNAi-mediated inhibition of CXCR7 partially suppressed HCC tumor growth in nude mice. Tumor angiogenesis is essential for both cancer growth and lethal metastatic cancer spread [35]. To investigate potential mechanisms underlying the CXCR7 silencing-mediated reduction in tumor growth, we examined the expression of gene (CD31) regulating angiogenesis in the tumors of mice. We found that inhibition of CXCR7 resulted in reduction in MVD. Thus, it is reasonable to speculate that inhibition of angiogenesis may lead to a significant delay in tumor growth. We did not observe that cancer cells spontaneously metastasize to other organs, such as lung, liver and spleen. Also, tumor metastasis was not affected after knockdown of CXCR7 expression in HCC cells. One possible reason is that SMMC-7721 cells are unable to metastasize to other organs by subcutaneous tansplantation in mice. Thus, we can not conclude that expression of CXCR7 do not affect tumor metastasis *in vivo*. Orthotopic implantation of HCC cells should be used to further evaluate the role of CXCR7 in regulating tumor metastasis.

The above findings imply that CXCL12/CXCR7 interaction may regulate multiple processes in HCC invasion and tumor growth. First, CXCR7 could affect CXCL12 induced tumor cell adhesion to ECM. Second, CXCR7 could regulate HCC invasive ability through angiogenesis and VEGF secretion. Third, up-regulation of CXCR7 expression by VEGF stimulation could enhance the invasive ability of cancer cells. Thus, we provide mechanistic evidence that CXCL12/CXCR7 interaction may affect HCC progression by multiple mechanisms including adhesion, invasion, angiogenesis, VEGF production and tumor growth. Because CXCR4 is also a receptor for CXCL12, we can not exclude the possibility that CXCR4 may be involved in regulating these biological behaviors triggered by CXCR7. Although our study shows the importance of CXCR7 in HCC invasion, angiogenesis and tumor growth, the role of CXCL12/CXCR7 interaction in tumor progression are not fully established. Moreover, a recent study has shown that AMD3100, a small synthetic inhibitor of CXCR4, not binds only to CXCR4, but also to CXCR7 [31]. We propose that more attention should be paid to CXCL12/CXCR4 axis and CXCL12/CXCR7 axis. Thus, further studies elucidating the role of CXCL12/CXCR7 axis in cancer development is needed.

## Conclusions

In summary, CXCR7 was highly expressed in hepatocellular carcinoma tissues. We presented the first evidence that suppression of CXCR7 expression by RNA interference impairs *in vitro *cellular invasion, adhesion, VEGF secretion and angiogenesis. We also observed that knockdown of CXCR7 significantly inhibited tumor growth but not metastasis *in vivo*. Moreover, we found that VEGF stimulation up-regulated the expression of CXCR7 in SMMC-7721 cells and HUVECs. Taken together, this study provides novel evidence that inhibition of CXCR7 expression may be an effective approach to suppressing tumor growth of HCC.

## Competing interests

The authors declare that they have no competing interests.

## Authors' contributions

KZ and GSR designed the experiments, KZ carried out most of experiments and drafted the manuscript. XYW and FL assisted with animal experiments. TT carried out cell culture of HUVECs. HYL and XLS participated in statistical analysis and interpretation of data. All authors read and approved the final manuscript.

## References

[B1] Mann CD Neal CP Garcea G Manson MM Dennison AR Berry DP Prognostic molecular markers in hepatocellular carcinoma: a systematic review Eur J Cancer 2007 43 6 979 92 10.1016/j.ejca.2007.01.004 17291746

[B2] Tung-Ping Poon R Fan ST Wong J Risk factors, prevention, and management of postoperative recurrence after resection of hepatocellular carcinoma Ann Surg 2000 232 1 10 24 10.1097/00000658-200007000-00003 PMC1421103 10862190

[B3] Müller A Homey B Soto H Ge N Catron D Buchanan ME McClanahan T Murphy E Yuan W Wagner SN Barrera JL Mohar A Verástegui E Zlotnik A Involvement of chemokine receptors in breast cancer metastasis Nature 2001 410 6824 50 6 10.1038/35065016 11242036

[B4] Miao Z Luker KE Summers BC Berahovich R Bhojani MS Rehemtulla A Kleer CG Essner JJ Nasevicius A Luker GD Howard MC Schall TJ CXCR7 (RDC1) promotes breast and lung tumor growth in vivo and is expressed on tumor-associated vasculature Proc Natl Acad Sci USA 2007 104 40 15735 40 10.1073/pnas.0610444104 PMC1994579 17898181

[B5] Pablos JL Amara A Bouloc A Santiago B Caruz A Galindo M Delaunay T Virelizier JL Arenzana-Seisdedos F Stromal-Cell Derived Factor Is Expressed by Dendritic Cells and Endothelium in Human Skin Am J Pathol 1999 155 5 1577 86 10.1016/S0002-9440(10)65474-0 PMC1866989 10550315

[B6] Aiuti A Webb IJ Bleul C Springer T Gutierrez-Ramos JC The Chemokine SDF-1 Is a Chemoattractant for Human CD34+ Hematopoietic Progenitor Cells and Provides a New Mechanism to Explain the Mobilization of CD34+ Progenitors to Peripheral Blood J Exp Med 1997 185 1 111 20 10.1084/jem.185.1.111 PMC2196104 8996247

[B7] Tashiro K Tada H Heilker R Shirozu M Nakano T Honjo T Signal sequence trap: a cloning strategy for secreted proteins and type I membrane proteins Science 1993 261 5121 600 3 10.1126/science.8342023 8342023

[B8] Murphy PM The molecular biology of leukocyte chemoattractant receptors Annu Rev Immunol 1994 12 593 633 10.1146/annurev.iy.12.040194.003113 8011292

[B9] Nagasawa T Hirota S Tachibana K Takakura N Nishikawa S Kitamura Y Yoshida N Kikutani H Kishimoto T Defects of B-cell lymphopoiesis and bone-marrow myelopoiesis in mice lacking the CXC chemokine PBSF/SDF-1 Nature 1996 382 6592 635 8 10.1038/382635a0 8757135

[B10] Marchesi F Monti P Leone BE Zerbi A Vecchi A Piemonti L Mantovani A Allavena P Increased Survival, Proliferation, and Migration in Metastatic Human Pancreatic Tumor Cells Expressing Functional CXCR4 Cancer Res 2004 64 22 8420 7 10.1158/0008-5472.CAN-04-1343 15548713

[B11] Sutton A Friand V Brulé-Donneger S Chaigneau T Ziol M Sainte-Catherine O Poiré A Saffar L Kraemer M Vassy J Nahon P Salzmann JL Gattegno L Charnaux N Stromal Cell-Derived Factor-1/Chemokine (C-X-C Motif) Ligand 12 Stimulates Human Hepatoma Cell Growth, Migration, and Invasion Mol Cancer Res 2007 5 1 21 33 10.1158/1541-7786.MCR-06-0103 17259344

[B12] Tachibana K Hirota S Iizasa H Yoshida H Kawabata K Kataoka Y Kitamura Y Matsushima K Yoshida N Nishikawa S Kishimoto T Nagasawa T The chemokine receptor CXCR4 is essential for vascularization of the gastrointestinal tract Nature 1998 393 6685 591 4 10.1038/31261 9634237

[B13] Zou YR Kottmann AH Kuroda M Taniuchi I Littman DR Function of the chemokine receptor CXCR4 in haematopoiesis and in cerebellar development Nature 1998 393 6685 595 9 10.1038/31269 9634238

[B14] Taichman RS Cooper C Keller ET Pienta KJ Taichman NS McCauley LK Use of the stromal cell-derived factor-1/CXCR4 pathway in prostate cancer metastasis to bone Cancer Res 2002 62 6 1832 7 11912162

[B15] Schimanski CC Bahre R Gockel I Müller A Frerichs K Hörner V Teufel A Simiantonaki N Biesterfeld S Wehler T Schuler M Achenbach T Junginger T Galle PR Moehler M Dissemination of hepatocellular carcinoma is mediated via chemokine receptor CXCR4 Br J Cancer 2006 95 2 210 7 10.1038/sj.bjc.6603251 PMC2360625 16819541

[B16] Kakinuma T Hwang ST Chemokines, chemokine receptors, and cancer metastasis J Leukoc Biol 2006 79 4 639 51 10.1189/jlb.1105633 16478915

[B17] Li YM Pan Y Wei Y Cheng X Zhou BP Tan M Zhou X Xia W Hortobagyi GN Yu D Hung MC Upregulation of CXCR4 is essential for HER2-mediated tumor metastasis Cancer Cell 2004 6 5 459 69 10.1016/j.ccr.2004.09.027 15542430

[B18] De Clercq E The bicyclam AMD3100 story Nat Rev Drug Discov 2003 2 7 581 7 10.1038/nrd1134 12815382

[B19] Burns JM Summers BC Wang Y Melikian A Berahovich R Miao Z Penfold ME Sunshine MJ Littman DR Kuo CJ Wei K McMaster BE Wright K Howard MC Schall TJ A novel chemokine receptor for SDF-1 and I-TAC involved in cell survival, cell adhesion, and tumor development J Exp Med 2006 203 9 2201 13 10.1084/jem.20052144 PMC2118398 16940167

[B20] Grymula K Tarnowski M Wysoczynski M Drukala J Barr FG Ratajczak J Kucia M Ratajczak MZ Overlapping and distinct role of CXCR7-SDF-1/ITAC and CXCR4-SDF-1 axes in regulating metastatic behavior of human rhabdomyosarcomas Int J Cancer 2010 10.1002/ijc.25245 PMC2907445 20162608

[B21] Zabel BA Wang Y Lewén S Berahovich RD Penfold ME Zhang P Powers J Summers BC Miao Z Zhao B Jalili A Janowska-Wieczorek A Jaen JC Schall TJ Elucidation of CXCR7-mediated signaling events and inhibition of CXCR4-mediated tumor cell transendothelial migration by CXCR7 ligands J Immunol 2009 183 5 3204 11 10.4049/jimmunol.0900269 19641136

[B22] Mazzinghi B Ronconi E Lazzeri E Sagrinati C Ballerini L Angelotti ML Parente E Mancina R Netti GS Becherucci F Gacci M Carini M Gesualdo L Rotondi M Maggi E Lasagni L Serio M Romagnani S Romagnani P Essential but differential role for CXCR4 and CXCR7 in the therapeutic homingof human renal progenitor cells J Exp Med 2008 205 2 479 90 10.1084/jem.20071903 PMC2271008 18268039

[B23] Iwakiri S Mino N Takahashi T Sonobe M Nagai S Okubo K Wada H Date H Miyahara R Higher expression of chemokine receptor CXCR7 is linked to early and metastatic recurrence in pathological stage I nonsmall cell lung cancer Cancer 2009 115 11 2580 93 10.1002/cncr.24281 19309748

[B24] Wang J Shiozawa Y Wang J Wang Y Jung Y Pienta KJ Mehra R Loberg R Taichman RS The Role of CXCR7/RDC1 as a Chemokine Receptor for CXCL12/SDF-1 in Prostate Cancer J Biol Chem 2008 283 7 4283 94 10.1074/jbc.M707465200 18057003

[B25] Maréchal R Demetter P Nagy N Berton A Decaestecker C Polus M Closset J Devière J Salmon I Van Laethem JL High expression of CXCR4 may predict poor survival in resected pancreatic adenocarcinoma Br J Cancer 2009 100 9 1444 51 10.1038/sj.bjc.6605020 PMC2694427 19352387

[B26] Meijer J Ogink J Roos E Effect of the chemokine receptor CXCR7 on proliferation of carcinoma cells in vitro and in vivo Br J Cancer 2008 99 9 1493 501 10.1038/sj.bjc.6604727 PMC2579699 18854833

[B27] Epstein RJ The CXCL12-CXCR4 chemotactic pathway as a target of adjuvant breast cancer therapies Nat Rev Cancer 2004 4 11 901 9 10.1038/nrc1473 15516962

[B28] Ruffini PA Morandi P Cabioglu N Altundag K Cristofanilli M Manipulating the chemokine-chemokine receptor network to treat cancer Cancer 2007 109 12 2392 404 10.1002/cncr.22706 17503430

[B29] Thelen M Thelen S CXCR7, CXCR4 and CXCL12: An eccentric trio? J Neuroimmunol 2008 198 1-2 9 13 10.1016/j.jneuroim.2008.04.020 18533280

[B30] Kalatskaya I Berchiche YA Gravel S Limberg BJ Rosenbaum JS Heveker N AMD3100 Is a CXCR7 Ligand with Allosteric Agonist Properties Mol Pharmacol 2009 75 5 1240 7 10.1124/mol.108.053389 19255243

[B31] Folkman J Angiogenesis in cancer, vascular, rheumatoid and other disease Nat Med 1995 1 1 27 31 10.1038/nm0195-27 7584949

[B32] Kijowski J Baj-Krzyworzeka M Majka M Reca R Marquez LA Christofidou-Solomidou M Janowska-Wieczorek A Ratajczak MZ The SDF-1-CXCR4 Axis Stimulates VEGF Secretion and Activates Integrins but does not Affect Proliferation and Survival in Lymphohematopoietic Cells Stem Cells 2001 19 5 453 66 10.1634/stemcells.19-5-453 11553854

[B33] Darash-Yahana M Pikarsky E Abramovitch R Zeira E Pal B Karplus R Beider K Avniel S Kasem S Galun E Peled A Role of high expression levels of CXCR4 in tumor growth, vascularization, and metastasis FASEB J 2004 18 11 1240 2 10.1096/fj.03-0935fje 15180966

[B34] Ferrara N VEGF and the quest for tumour angiogenesis factors Nat Rev Cancer 2002 2 10 795 803 10.1038/nrc909 12360282

[B35] Folkman J What is the evidence that tumors are angiogenesis dependent? J Natl Cancer Inst 1990 82 1 4 6 10.1093/jnci/82.1.4 1688381

